# Supplementary figures and images for: Cytogenetic Mapping of 35 New Markers in the Alpaca (Vicugna pacos)
Source: Genes (Basel). 2020 May 8;11(5):522. doi: 10.3390/genes11050522 (PMC7288448; doi:10.3390/genes11050522)

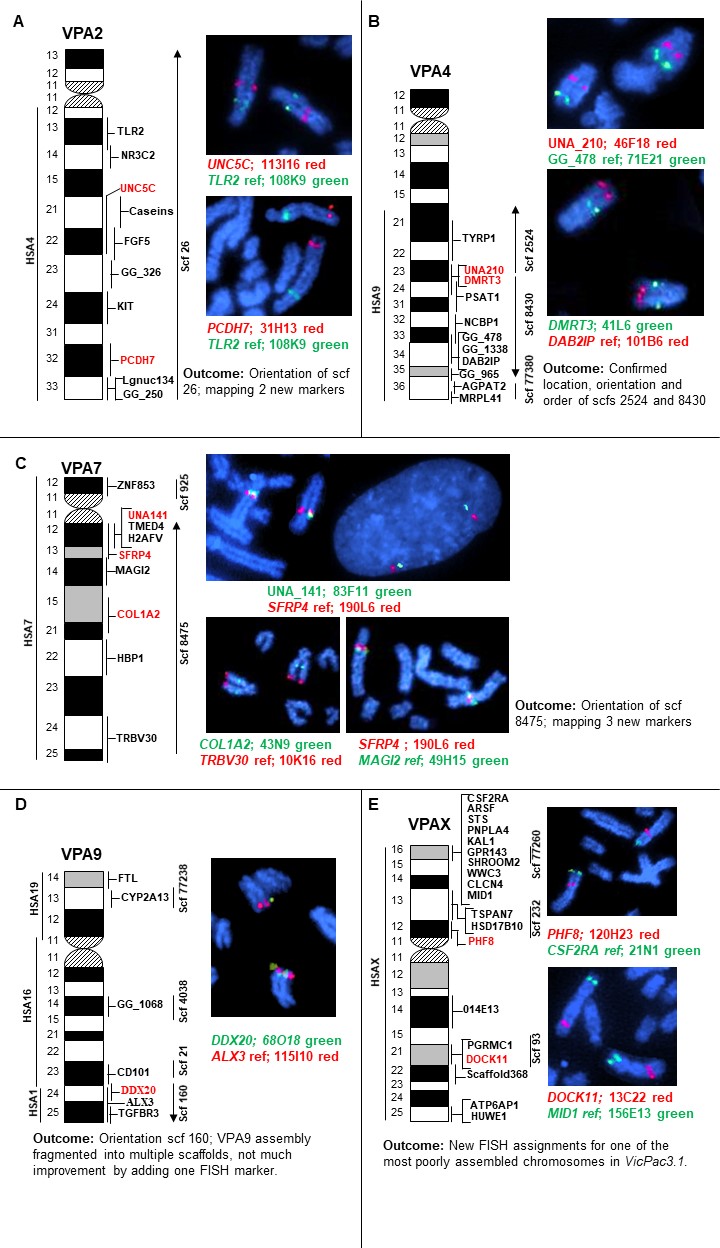

Supplement: Supplementary file 1 [file genes-11-00522-s001.zip › Supplementary material_Submission II_R1/Supplementary Figure S1_FISH_Final I/Slide1.JPG]

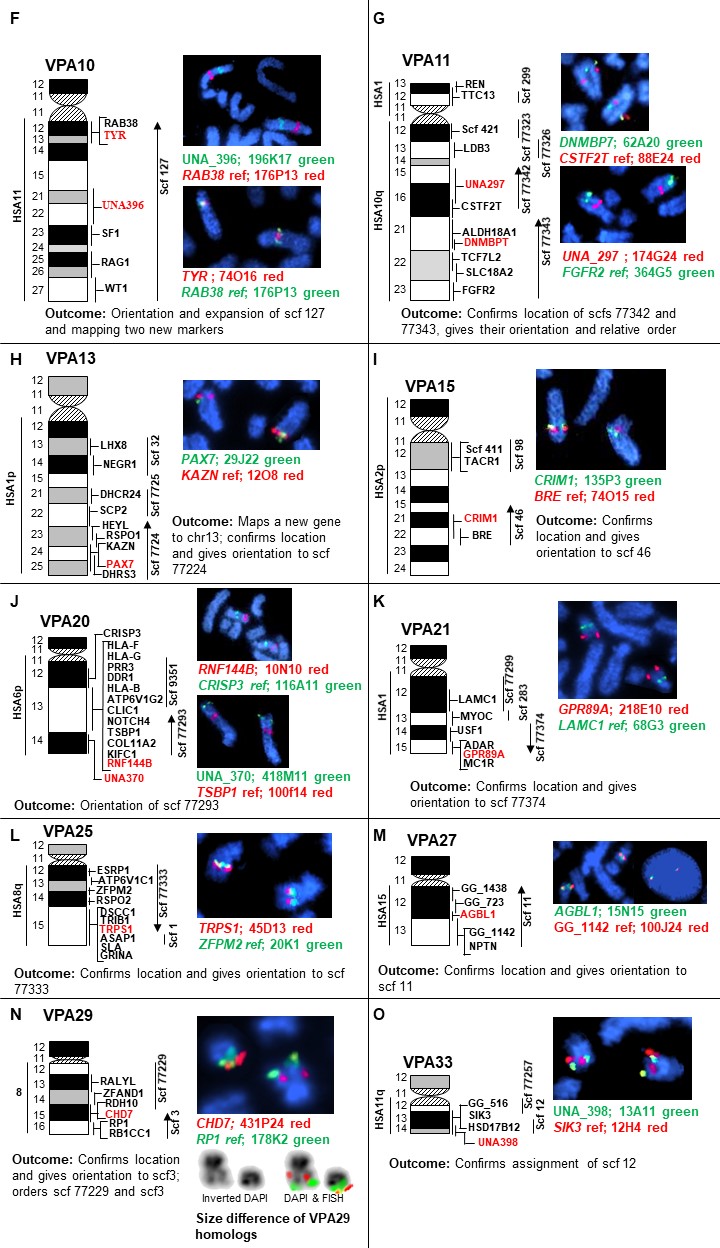

Supplement: Supplementary file 1 [file genes-11-00522-s001.zip › Supplementary material_Submission II_R1/Supplementary Figure S1_FISH_Final I/Slide2.JPG]

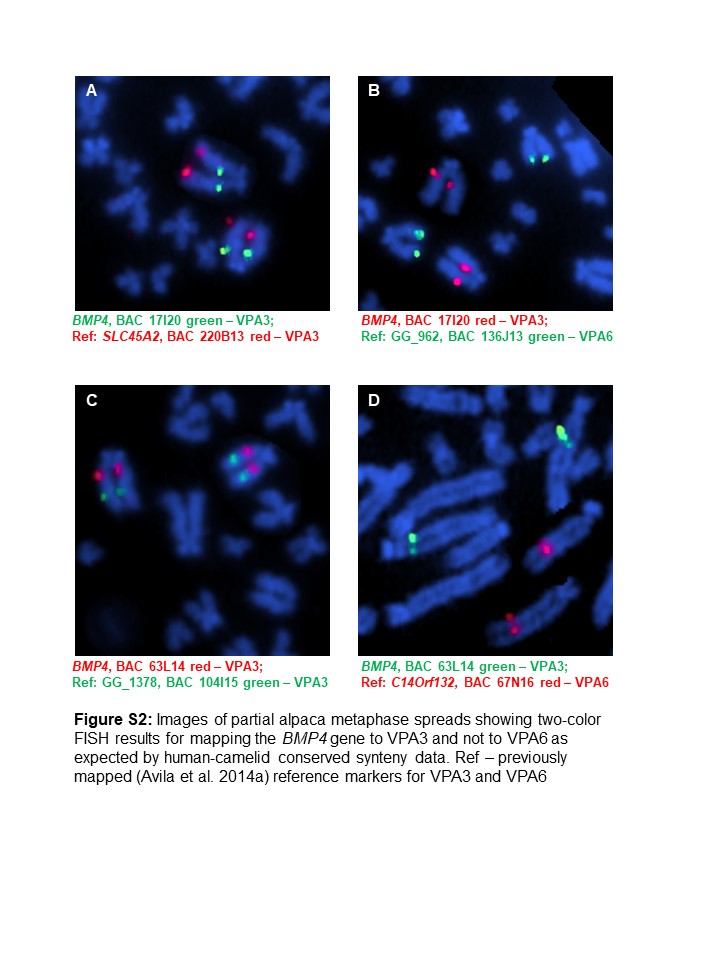

Supplement: Supplementary file 1 [file genes-11-00522-s001.zip › Supplementary material_Submission II_R1/Supplementary Figure S2_BMP4_Final I.jpg]
